# Recognition Memory Induces Natural LTP-like Hippocampal Synaptic Excitation and Inhibition

**DOI:** 10.3390/ijms231810806

**Published:** 2022-09-16

**Authors:** Irene Sánchez-Rodríguez, Sara Temprano-Carazo, Danko Jeremic, Jose Maria Delgado-Garcia, Agnès Gruart, Juan D. Navarro-López, Lydia Jiménez-Díaz

**Affiliations:** 1 Neurophysiology & Behavior Laboratory, Regional Centre for Biomedical Research (CRIB), School of Medicine of Ciudad Real, University of Castilla-La Mancha, 13071 Ciudad Real, Spain; 2 Division of Neurosciences, University Pablo de Olavide, 41013 Seville, Spain

**Keywords:** synaptic plasticity, excitatory/inhibitory LTP, GirK, hippocampus, freely moving mice, recognition memory

## Abstract

Synaptic plasticity is a cellular process involved in learning and memory by which specific patterns of neural activity adapt the synaptic strength and efficacy of the synaptic transmission. Its induction is governed by fine tuning between excitatory/inhibitory synaptic transmission. In experimental conditions, synaptic plasticity can be artificially evoked at hippocampal CA1 pyramidal neurons by repeated stimulation of Schaffer collaterals. However, long-lasting synaptic modifications studies during memory formation in physiological conditions in freely moving animals are very scarce. Here, to study synaptic plasticity phenomena during recognition memory in the dorsal hippocampus, field postsynaptic potentials (fPSPs) evoked at the CA3–CA1 synapse were recorded in freely moving mice during object-recognition task performance. Paired pulse stimuli were applied to *Schaffer* collaterals at the moment that the animal explored a new or a familiar object along different phases of the test. Stimulation evoked a complex synaptic response composed of an ionotropic excitatory glutamatergic fEPSP, followed by two inhibitory responses, an ionotropic, GABA_A_-mediated fIPSP and a metabotropic, G-protein-gated inwardly rectifying potassium (GirK) channel-mediated fIPSP. Our data showed the induction of LTP-like enhancements for both the glutamatergic and GirK-dependent components of the dorsal hippocampal CA3–CA1 synapse during the exploration of novel but not familiar objects. These results support the contention that synaptic plasticity processes that underlie hippocampal-dependent memory are sustained by fine tuning mechanisms that control excitatory and inhibitory neurotransmission balance.

## 1. Introduction

Santiago Ramón y Cajal is generally acknowledged as the father of the idea that the brain is made up of neurons as the physiological signal units of the brain (neuron doctrine). More remarkably, he was eager to speculate from his anatomical artistic imagery that the synapses provide the opportunity for modification by experience (cerebral gymnastics) [1,2]. The same idea lies at the core of contemporary investigations based on the synaptic plasticity hypothesis, which posits that the ease with which a signal in one cell excites (or inhibits) its target cell is not fixed but modifiable (i.e., plastic) [3,4,5]. Bliss and Lømo’s discovery that the formation of memories from short-term into long-term may largely involve coded strengthening of hippocampal synapses—what is called long-term potentiation (LTP)—paved the way to study how experience modifies synaptic strength in hippocampus and other brain regions [6,7,8,9,10,11].

LTP is the learning-related molecular mechanism best described to date that acts as a functional correlate of memory storage [9,11,12]. The experience may strengthen synaptic connections, or weaken them, a process named long-term depression (LTD), so that synaptic plasticity is a dynamic and bidirectional process. Additionally, short-term mechanisms were identified, and studies showed that the activity at one synapse on a CA1 neuron can increase the sensitivity of that synapse to further change even without inducing plasticity [13,14,15,16,17]. The brain, and the memory it uses, is ever changing and adapting and has its own homeostatic plasticity and “scaling” mechanisms of stabilizing excitability of neurons, preventing runaway plasticity, so the equilibrium between excitatory/inhibitory synaptic input is necessary for correct LTP induction [18]. Inhibitory neurotransmission mediated by G-protein-gated inwardly rectifying potassium (GirK) channels seems to have an important role in such balance maintenance and provides a way for neuromodulators (e.g., serotonin, adenosine, GABA_B_, and hormones) to regulate the excitability of neurons by hyperpolarizing the resting potential [19,20,21] and by decreasing the amplitude of EPSPs via shunting inhibition mechanisms [22,23,24].

In synaptic plasticity studies, the stimulus that modified synapses is generally electrical stimulation. Memories, however, are established as a result of a behavioral experience, where the behaving organism interacts with its environment [6]. LTP has been widely studied in glutamatergic synapses, and it has also been described for inhibitory neurotransmission in vitro [25,26,27,28,29] and recently in vivo [21,30], but it has always been evoked experimentally by the application of an induction stimulating protocol (i.e., high-frequency stimulation (HFS), theta burst stimulation (TBS), and spike-timing dependent plasticity (STDP)). So far, a limited number of studies have been conducted examining the induction of long-term synaptic modifications during memory formation in physiological conditions, without any artificial induction protocol [31]. Here, we explore whether memory-induced changes in the CA3–CA1 synapse that facilitate the retrieval of new recognition memories (memory-induced LTP-like event) would be necessary in freely-moving mice during the novel-object recognition (NOR) task.

## 2. Results

**Figure 1:**
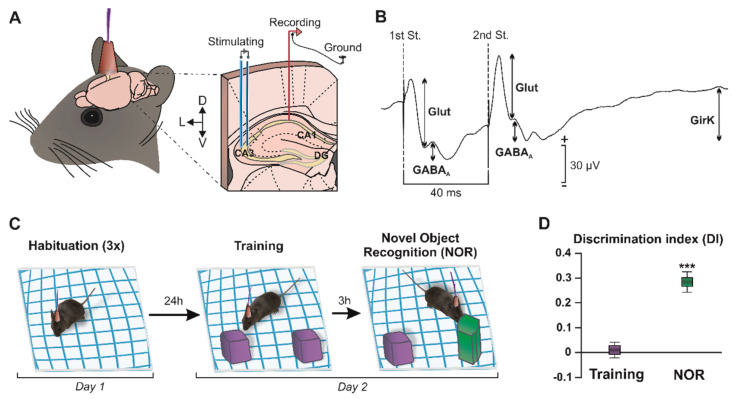
Experimental design. (**A**) The picture illustrates how mice were prepared for chronic recording of fPSPs in the hippocampal CA1 region, by surgical implantation of bipolar stimulating electrodes on the right Schaffer collaterals, and bipolar recording electrodes at ipsilateral CA1. A bare silver wire was fixed to the skull as ground. (**B**) Representation of the fPSPs evoked in the CA1 hippocampal region after paired-pulse stimulation (40-ms interstimulus interval) at the *Schaffer* collaterals. The representative recording illustrates the averaged (n = 50) profile of the postsynaptic response. For each fPSP, the maximum amplitude (peak-to-peak value, see arrows) was measured for the analysis. DG, dentate gyrus; St., stimulus; D, dorsal; L, lateral; V, ventral; and Glut, glutamate. (**C**) NOR task. The object recognition protocol consisted of three 5-min habituation sessions with the empty box (interval of 1.5 h between the habituations) on day 1. On day 2, two identical objects were placed (purple cubes) in the center of the box and the animals (n = 36) were allowed to explore them for 10 min (training session). Three hours later, one of the objects was replaced by a novel one (green prism) for the NOR session. (**D**) Graph representing the discrimination index (DI) during the training (two identical objects) and NOR (familiar vs. novel object) sessions. DI = 0, no discrimination between objects. *** *p* < 0.001 vs. DI = 0.

As detailed in the Methods section, animals were prepared for the chronic recording of field postsynaptic potentials (fPSPs) at hippocampal CA3–CA1 synapses (Figure 1A,B) in freely movement (Figure 1C), as previously described [21,32]. The electrical stimulation of *Schaffer* collaterals evoked a complex synaptic response in the CA1 pyramidal cells with three different components (Figure 1B): an excitatory glutamatergic fEPSP, with a latency of appearance of 2.25–4 ms after stimulation followed by a GABA_A_ receptors-dependent fIPSP; with a latency of 12–15 ms; and, finally, a delayed fIPSP with a latency of 26–36 ms, generated by an increase in potassium conductance (efflux) via GirK channels [21].

### 2.1. Development of Recognition Memory during a NOR Task

Recognition memory is a cognitive capability that significantly relies on the correct functionality of the CA3–CA1 synapse in the dorsal hippocampus [31,32]. The NOR test was performed to address whether mice showed object memory retention. In the training session (Figure 1C), where two identical objects were presented to the subjects, animals (n = 36) spent a similar amount of time exploring each object, which resulted in a discrimination index (DI) of approximately zero, indicating the absence of discrimination or preference between objects (Figure 1D; DI = 0.01 ± 0.03, *t*(35) = 0.33, *p* = 0.746). In contrast, during the NOR session, which took place 3 h after training, mice showed a marked preference for the exploration of the novel object (Figure 1D, DI = 0.28 ± 0.04, *t*(35) = 6.88, *p* < 0.001), showing that long-term memory remained intact in these animals. These results indicate that the synaptic plasticity processes underlying the establishment and consolidation of memories (such as LTP in the hippocampal synapse CA3–CA1) [17] were likely functional in vivo in these animals.

### 2.2. Object Recognition Induces Synaptic Plasticity Changes in the Hippocampus

**Figure 2:**
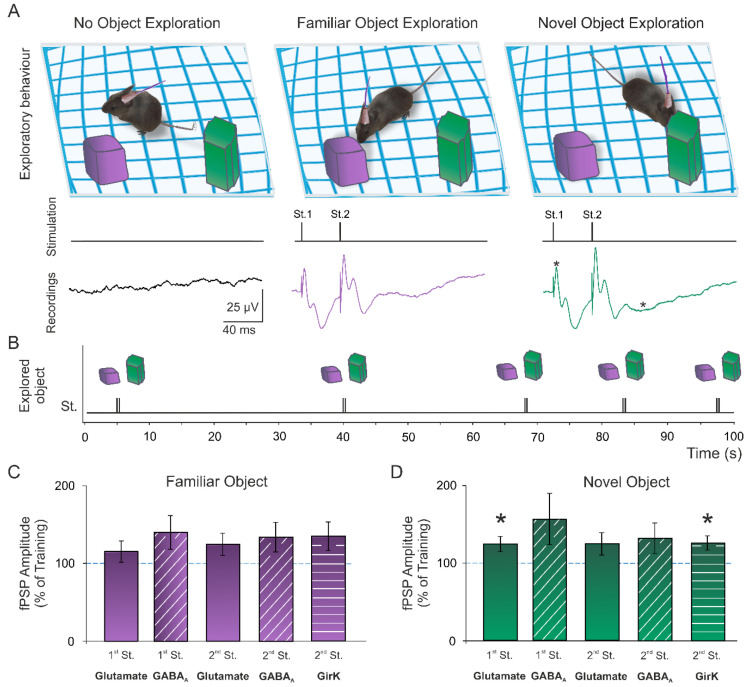
Memory-induced synaptic plasticity. (**A**) During the novel object recognition (NOR) session, animals were stimulated in the *Schaffer* collaterals with paired pulses (40-ms interstimulus interval) when displaying an exploratory behavior towards either the familiar or novel object. (**B**) Pulses were applied in alternate exploratory events, with a minimum of 10 s between stimulation. The response evoked in CA1 was recorded for amplitude analysis. (**C**,**D**) Graphs represent the mean ± standard error of the amplitude of fEPSPs and fIPSPs evoked in the CA1 area by electrical stimulation during the NOR session when mice explored the familiar ((**C**); n = 21) and the novel object ((**D**); n = 22). All data were normalized as a percentage of the values recorded during the training session. *, *p* < 0.05 vs. training (100%). St., stimulus.

During training and NOR sessions, an external stimulator was used to apply paired pulses in the *Schaffer* collaterals when animals explored either of the objects. Pulses were applied in alternate exploratory events, with a minimum of 10 s between stimulation (Figure 2A,B; see details in the methods section). fPSP amplitudes were measured (n = 21–22) and normalized to the training session values (100%) (Figure 2C,D). Then, a significant potentiation of the glutamatergic fEPSP evoked by the 1st stimulus during the NOR test (*t*(20) = 2.11, *p* = 0.048) when animals explored the novel object could be detected, but not the familiar one (Figure 2C,D; *t*(21) = 1.27, *p* = 0.22). In the same manner, an increase in the GirK-dependent component was induced only when the novel object (but not the familiar one) was being explored (Figure 2C,D; *t*(20) = 2.25, *p* = 0.036). These data show a differential excitatory and inhibitory synaptic plasticity depending on the familiarity of the presented objects, which might explain the object discrimination observed when analyzing the exploration times.

## 3. Discussion

The hippocampus contains a modifiable synapsis that provides the acquisition of declarative memory [33], including episodic, semantic, and familiarity-based recognition memory [34], and spatial learning and memory in animals [35,36] and humans [37,38]. Given that hippocampal pyramidal neurons have >10,000 independently modifiable synapses, the potential for information storage by synaptic modification, neuromodulation, and other priming events is enormous [39]. Evidence presented by anatomical, neurophysiological, and behavioral studies reveals differences in the hippocampus along the dorsoventral axis [40]. It has been reported that the disturbance of either dorsal or ventral hippocampal neurons causes cognitive deficits; therefore, both regions would be essential for the acquisition and retrieval of hippocampal-dependent memories [41]. However, some authors highlight the participation of specific hippocampal regions depending on the type of learning that is taking place [42,43,44]. The main consensus defends the participation of the dorsal hippocampus in space navigation and memory, while the ventral hippocampus would play a main role in anxiety behaviors [40,45], although a role in memory cannot be ruled out [46]. Our findings support the idea that memory-induced LTP-like events are taking place in the dorsal hippocampus, from where recordings were obtained in freely-moving animals, during object-recognition memory retrieval. However, the possibility that synaptic changes may also occur in the ventral hippocampus cannot be ruled out with our experimental approach [46,47].

On the other hand, it is worth noting the relevance of the complex response obtained in the experiments performed here. Such fPSP has previously been described in different regions where the balance between excitatory and inhibitory neurotransmission underlay superior functional roles. For example, ex vivo, it has been found in pyramidal neurons of basolateral amygdaloid nucleus [48], or in CA3 pyramidal cells after hilus and mossy fibers stimulation [49,50]. Ex vivo, we also found it in CA3 pyramidal neurons after fimbria stimulation, and using intracellular recordings with sharp electrodes we could perform its pharmacological dissection, comprising the same three phases: an ionotropic glutamatergic EPSP followed by two IPSPs, early (GABA_A_), and late (GABA_B_) [51]. However, although it could also be identified in vivo at CA3–CA1 synapse [52,53], this response is much more difficult to induce. Therefore, in order to show more clearly the different components of the complex response in freely-moving mice, we used paired, instead of single, pulses in our experiments, as paired-pulse facilitation (PPF) is a characteristic of physiological CA3–CA1 synapse. Then, this protocol led to the enhancement of all components of the complex response, including the late negative components presented in the fPSPs, which have been shown to correspond to the activation of GABA_A_ and GIRK [21,52]. In addition, the GirK component must be analyzed in the second response as its latency of appearance is 26–36 ms and interval of paired-pulse is 40 ms. Due to the recent identification of the critical role of GirK channels for synaptic plasticity in the dorsal hippocampus ex vivo [54] and in vivo [19], and knowing that different neurotransmission systems (GABAergic, adenosinergic, dopaminergic, opioid…) have GirK channels as main effector in the CA1 region of dorsal hippocampus [20,55], it seems necessary to more deeply explore the role of GirK in learning and memory processes.

### 3.1. Long-Term Recognition and Dorsal Hippocampus

The connections or mechanisms involved in recognition memory are not fully known yet, but it has been reported that temporary or permanent lesion of the hippocampus impairs object memory [56,57]. A large number of data from human and animal studies using psychological, electrophysiological, imaging, and lesion techniques indicates that the medial temporal lobe is crucial for recognition memory and more complex aspects of such a process, including recollective, contextual, associative, and spatial characteristics of recognition memory relying on the hippocampus [6,32,58,59,60,61,62,63]. In addition, both gain- or loss-of-function of GirK channel activity in the hippocampus have been shown to be deleterious for recognition memory [19] as they regulate neural excitability playing a crucial role in LTP/LTD threshold regulation [32,64] and in the induction and maintenance of plasticity processes [21]. Thus, the hippocampal circuit plays a significant role during this learning test. However, considerable debate has focused on whether this structure plays a significant role in the object memory encoded, consolidated and retrieved during discrete stages of the NOR task [57]. Some authors argue that, although the perirhinal cortex has been associated with the short-term recognition of objects (~1 h), the hippocampus is the structure responsible for long-term recognition (~24 h) [58,65,66]. In this sense, a delay-dependent role of the hippocampus in NOR has been shown within the framework of the medial temporal lobe, stating that temporary or permanent lesions of the hippocampus disrupt object memory when a delay ≥ 10 min exists between the training and test sessions [57]. In our experiments, the NOR test session took place 3 h after training; therefore, some contribution of dorsal hippocampus would be necessary for memory retention and retrieval.

### 3.2. Importance of Novelty in Memory-Induced Synaptic Potentiation

Control of excitability in neuronal membranes and synaptic plasticity mechanisms are essential for memory formation, and learning can induce changes in intrinsic excitability to facilitate the encoding of new memories [31,39,67,68]. The proper functionality of the CA3–CA1 hippocampal synapse is required for episodic, spatial, contextual, and recognition memory [57,69,70,71]. In particular, object recognition memories rely, among other events, on the adequate signaling of these neurons in the CA3–CA1 region [31,63]. The object-recognition test is a widely accepted task for the evaluation of non-spatial memory in rodents. This task is based on the natural tendency of these animals to explore novel objects for longer periods of time than familiar objects [72]. This preference for the novel object implies that the familiar object exists in the animal’s memory [58]. Although the hippocampus may not play a direct role in discriminating distinctive features of the different objects used for this test, it is essential for the detection of novelty due to its role in comparing the current situation with previously stored information [31]. The results of our behavioral test allowed us to verify that our subjects were able to incorporate the characteristics of an explored object to their memory and to tell it apart from a novel object hours later, and that the latter induced plastic changes of excitatory (glutamatergic) and inhibitory (GirK-mediated) activity.

Reconsolidation is a phase of retrieval and consolidation of memories when animals are presented with novelty. In this state, previously formed memories become labile and require stabilization to persist. The LTP process is involved in all phases of non-spatial hippocampal memories, such as object-recognition memory, including reconsolidation [31,58]. In this context, we previously reported a deficit in object recognition present in mice that had been treated to induce impairment of hippocampal LTP [19,32,73]. To explore the possible appearance of a natural memory-induced synaptic potentiation (that is, without the presentation of any electrical stimulation protocol for its induction) during this NOR task, an analysis of the amplitude of the excitatory and inhibitory fPSPs evoked in the CA1 region was performed. In this sense, in the NOR test session, 3 h after training, when animals showed exploratory behavior towards the novel object, we detected an enhancement in the amplitude of the glutamatergic fEPSP evoked by the first applied pulse, and the GirK-dependent fIPSP, when compared to the amplitudes of the same fPSPs during training. This synaptic potentiation could underlie the detection of novelty and the incorporation of this new object and its distinct characteristics to the animal’s memory, as no object-induced LTP was observed during the exploration of the familiar object.

Similarly, natural training-induced LTP of the fEPSPs at CA3–CA1 synapse, several hours after the training session of a NOR protocol, has been previously reported [31]. This LTP appeared without the need to present any object to the animals, (i.e., when training had been completed), which may indicate this form of plasticity is involved not only in the detection of the object’s characteristics during memory formation (encoding) but later for consolidation. In addition, and in agreement with our results, Clarke et al. [31] detected fEPSP potentiation in the NOR test session, which was not present during training. In that case, LTP was found when exploring both objects placed in the arena (familiar and novel), so LTP seemed to be related to the retrieval, during this reconsolidation stage, of the object memory previously formed in the training session. Our stimulation protocol (pulses applied during exploratory events) and the one used in that study (with additional recording sessions between exploratory tasks) differ. Our analysis to detect changes in synaptic activity efficacy was also different as we recorded baseline synaptic activity during pulses application in the training session instead of obtaining it during habituation (i.e., without any objects present). These facts might explain why we specifically detected memory-induced LTP differences depending on the novelty of the objects, i.e., when the animals were exploring the new object during memory retention assessment. As we compared to fPSPs evoked by stimulation during object exploration in the training phase, the exploration of the novel object induced an excitatory and inhibitory synaptic activity enhancement when compared to training, but the exploration of the familiar object during retrieval did not induce any plastic changes, as had also been shown during re-training with two familiar objects (no LTP-induction) [31].

Other studies have related exposure to novelty (either novel objects or familiar objects in a new location or context) with the facilitation of LTD and only detected the facilitation of LTP when animals explored a new environment, in the absence of objects, although they evaluated potentiation levels throughout the whole test rather than at exploratory events, which could mask object-dependent changes in the amplitude of postsynaptic potential [74]. In any case, Clarke et al. indeed found that NOR memory assessment induced an early depotentiation that could very likely be due to the natural GirK-mediated inhibitory LTP-like activity induced by NOR retrieval that we have found here for the first time. In fact, the presence of an inhibitory GirK-dependent LTP with a late appearance 48 h after an artificial HFS protocol has been previously demonstrated [21], suggesting that the plasticity of GirK channel signaling might be involved in the extinction of the fEPSP potentiation to basal amplitude levels [26,75]. There are factors that decrease the probability of reconsolidation taking place (age, sleep, memory strength, weak reactivation sessions, and predictable reactivated stimulus), as well as factors that promote reconsolidation (epigenetic priming, new information during reactivation, increased intensity of reactivation session, and plasticity enhancer strategies) [76,77,78]. In this sense, the modulation of GirK channels might provide new ways to promote memory reconsolidation.

In summary, our present results showed the presence of excitatory (glutamate-mediated) and, for the first time, inhibitory (GirK-mediated) learning-induced LTP at the hippocampal CA3–CA1 synapse during the exploration of novel but not familiar objects, supporting the contention that synaptic plasticity processes that underlie hippocampal-dependent memory retrieval are sustained by fine tuning mechanisms that control excitatory and inhibitory neurotransmission balance [19].

## 4. Materials and Methods

### 4.1. Subjects

C57BL/6 male adult mice (3–5 months old; 28–35 g; n = 40) obtained from an official supplier (Janvier Labs, Marseille, France) were used for electrophysiological and behavioral experiments. The general condition of animals was assessed on the day before and on the day of surgery. No signs of abnormalities were detected that could cause anatomical or behavioral alteration impacting the experimental results (scratches, bite marks, weight loss, unusual posture, etcetera). Following surgeries, animals were housed on a 12 h light/dark cycle with constant ambient temperature (21 ± 1 °C) and humidity (50 ± 7%) conditions. Food and water were accessible *ad libitum*. All experiments were performed in accordance with European Union guidelines (2010/63/EU) and with Spanish regulations for the use of laboratory animals in chronic experiments (RD 53/2013 on the care of experimental animals: BOE 08/02/2013) and approved by the local Ethics Committees of the Universities of Castilla-La Mancha and Pablo de Olavide.

### 4.2. Surgery

Stereotactic surgery took place to implant intra-hippocampal electrodes for electrical stimulation during behavioral tests, as well as the recording of CA3–CA1 synaptic activity. Surgeries were performed following previously described procedures [21]. Briefly, animals were anesthetized with 4–1.5% isoflurane (induction and maintenance, respectively; #13400264, ISOFLO^®^, Proyma S.L., Ciudad Real, Spain) delivered using a calibrated R580S vaporizer (RWD Life Science, Dover, DE, USA; flow rate: 0.5 L/min oxygen) and placed in a stereotaxic frame. Buprenorphine was administered intramuscularly as an analgesic during and after surgery (0.01 mg/kg; # 062009, BUPRENODALE^®^, Albet, Barcelona, Spain). Small orifices were created in the skull at appropriate coordinates (Paxinos and Franklin, 2001) to access the right hemisphere. A stimulating electrode was directed to the *Schaffer* collaterals via commissural of the dorsal hippocampus (2 mm lateral and 1.5 mm posterior to bregma, depth from the surface of the brain, 1.0–1.5 mm), and a recording electrode was aimed to the ipsilateral *stratum radiatum* under the pyramidal area of CA1 (1.2 mm lateral and 2.2 mm posterior to bregma, depth from the surface of the brain, 1.0–1.5 mm) (Figure 1A). A bare silver wire (0.1 mm) was affixed to the skull as a ground. The electrodes and the ground were soldered to a 6-pin socket, and all elements were fixed to the skull of the animal with dental cement.

At the end of the surgical procedure, the animal was placed in the recovery zone on absorbent paper in a cage, under a heating lamp, allowing the operator to proceed with an operation on a second animal while monitoring the recovery of the first. The animals were returned to the housing facilities after complete awakening from anesthesia.

### 4.3. Novel Object Recognition (NOR) Test

The NOR paradigm assesses an animal’s innate ability to differentiate an old from a new object. NOR experiments were performed under a dim light (30–40 lx) in an open field arena (30 × 25 × 20 cm) made of transparent polyvinyl chloride (PVC). Validated objects (to avoid default intrinsic preference) were statically fixed to the base of the arena, with enough separation from each other and the perimeter of the cage so that animals could surround each object. Both the box and the objects were cleaned between tests with water and soap and 70% alcohol to remove olfactory clues. Exploration was defined as sniffing or touching objects with the nose and/or front legs or directing the nose towards the object from less than 1 cm. Sitting on objects and/or walking around them was not considered exploratory behavior. All sessions were recorded with a video camera.

The NOR task consisted of three 5 min habituation sessions, on the first day of the experiment, in which animals were allowed to freely explore the open field arena in the absence of objects (Figure 1C). The next day, two 10-min tests took place (Figure 1C). Firstly, a training session was performed using two identical objects (two Lego^®^ purple cubes). Then, 3 h after training, one of the objects was replaced by a novel object (green prism) and a new exploration session took place to evaluate the retention of long-term memory (Figure 1C). The discrimination index (DI) was calculated for learning evaluation. It is defined as the difference of exploration time between the two objects (TO1 − TO2), divided by total exploration time (TO1 + TO2). That is, DI = (TO1 − TO2) / (TO1 + TO2). Only data from the animals that showed successful learning (~90% of the subjects), that is, those that explored the novel object for a significantly longer time than the familiar (Figure 1D), were analyzed.

Electrophysiological recordings were obtained during object-recognition tasks, and 100 μs square, biphasic pulses were applied in pairs, at an inter-stimulus interval of 40 ms and at the intensity needed to evoke ~35% of the maximum fEPSP response in the CA1 hippocampal region. Pulse pairs were manually applied in alternate exploratory events (minimum stimulation interval of 10 s) when the mice showed exploratory behavior towards either of the two objects present in the arena (Figure 1A,B).

Amplitudes of three different components of the response generated in CA1 were analyzed using the Spike2 and Signal software (Cambridge Electronic Design, Cambridge, UK). Each postsynaptic potential was identified by its latency of apparition, as previously described [21]: (1) a glutamatergic fEPSP, with a latency of appearance of 2.25–4 ms after stimulation; (2) a GABAergic fIPSP dependent on GABA_A_ receptors, with a latency of 12–15 ms; and (3) an fIPSP dependent on metabotropic receptors and GirK channels, with a latency of 26–36 ms (Figure 1B). The amplitudes evoked in each mouse during the NOR session were normalized as a percentage of the amplitudes at the training session.

### 4.4. Analysis and Statistics

Recordings were stored on a computer using an analog/digital converter (CED 1401 Plus). Data were analyzed offline for the measurement of fPSP amplitude using the Signal program (Cambridge Electronic Design, Cambridge, UK). The electrical recordings selected for analysis presented clear fPSPs, and their quality was maintained throughout the experiment days. Results were processed for graphic representation with SigmaPlot v11.0 (Systat Software, Palo Alto, CA, USA) and CorelDraw (v18, Corel Corporation, Ottawa, Canada). Data are represented as the mean ± standard error. All statistical calculations were performed using the SPSS Statistics software (v.28, SPSS Inc., New York, NY, USA). For learning and synaptic plasticity analysis, a one sample *t*-test was performed for comparisons (DI vs. no-discrimination (0%), fPSP amplitude vs. training (100%)). Statistical significance was established at *p* < 0.05.

## Abbreviations

fPSP	field postsynaptic potential
fEPSP	field excitatory postsynaptic potential
fIPSP	field inhibitory postsynaptic potential
GirK	G-protein-gated potassium channels
TBS	theta burst stimulation
HFS	high-frequency stimulation
STDP	spike-timing dependent plasticity
LTD	long-term depression
LTP	long-term potentiation
NOR	novel object recognition
DI	discrimination index
TO	time in object
PPF	paired pulse facilitation
